# Seed treatment with prodigiosin controls damping-off of cucumber caused by *Pythium ultimum*

**DOI:** 10.1186/s13568-020-01169-2

**Published:** 2021-01-06

**Authors:** Daniel P. Roberts, Kaitlyn Selmer, Robert Lupitskyy, Clifford Rice, Jeffrey S. Buyer, Jude E. Maul, Dilip K. Lakshman, Jorge DeSouza

**Affiliations:** 1 Sustainable Agricultural Systems Laboratory, USDA-Agricultural Research Service, Beltsville Agricultural Research Center, BLDG 001, Rm. 245B, Beltsville, MD 20705 USA; 2 Departamento de Fitopatologia, Universidade Federal de Lavras, Lavras, 37200 Brazil; 3 Present Address: Agios Pharmaceuticals, 88 Sidney St, Cambridge, MA USA; 4 Present Address: TIC Gums, 10552 Philadelphia Rd., White Marsh, MD 21162 USA

**Keywords:** Natural product, Prodigiosin, *Pythium ultimum*, Serratamolides, *Serratia marcescens*, Serrawettin

## Abstract

Ethanol extract of cell mass of *Serratia marcescens* strain N4-5, when applied as a treatment to cucumber seed, has been shown to provide control of the oomycete soil-borne plant pathogen *Pythium ultimum* equivalent to that provided by a seed-treatment chemical pesticide in some soils. Two dominant compounds in this extract, prodigiosin and the serratamolide serrawetin W1, were identified based on mass and collision induced dissociation mass fragmentation spectra. An additional four compounds with M+H^+^ masses (487, 541, 543, and 571) consistent with serratamolides reported in the literature were also detected. Several other compounds with M+H^+^ masses of 488, 536, 684, 834, 906, and 908 *m/z* were detected in this ethanol extract inconsistently over multiple liquid chromatography coupled with tandem mass spectrometry (LC/MS–MS) runs. A purified preparation of prodigiosin provided control of damping-off of cucumber caused by *P. ultimum* when applied as a seed treatment while ethanol extract of cell mass of strain Tn246, a transposon-mutant-derivative of strain N4-5, did not. Strain Tn246 contained a mini-Tn5 Km insertion in a prodigiosin biosynthetic gene and was deficient in production of prodigiosin. All other compounds detected in N4-5 extract were detected in the Tn246 extract. This is the first report demonstrating that prodigiosin can control a plant disease. Other compounds in ethanol extract of strain N4-5 may contribute to disease control.

## Key points

- Ethanol extract of *S. marcescens* N4-5, containing prodigiosin and serratamolides, controls damping-off of cucumber caused by *Pythium ultimum.*
- Purified prodigiosin controlled damping-off of cucumber when applied as a seed treatment.
- Ethanol extract of a transposon mutant of *S. marcescens* N4-5 did not contain prodigiosin and did not control damping-off when applied as a seed treatment.

## Introduction

The oomycete *Pythium ultimum* Trow is an important soil-borne plant pathogen that causes damping-off and other diseases on over 300 diverse plant species including cucumber and other cucurbits (Kamoun et al. 2014; Okubara et al. 2014). New control measures are needed for this pathogen and related oomycetes as existing controls can be problematic. For example, cultural methods such as crop rotation are sometimes ineffective due to the long-term persistence in soil and wide host range of *P. ultimum*. Seed treatment with pesticides, especially mefenoxam when available, can be very effective for managing damping-off diseases caused by this pathogen (Garzón et al. 2011), but there are concerns regarding the development of resistance in pathogen populations (Lamour and Hausbeck 2000; Moorman and Kim 2004; Okubara et al. 2014; Taylor et al. 2002).

Natural products are being considered for disease control as they provide new modes of action or serve as lead structures for modification for development of new chemistries (Cantrell et al. 2012; Dayan et al. 2009; Gerwick and Sparks 2014; Hüter 2011; Rutledge and Challis 2015). Natural products can also be used for disease control in organic cropping systems while synthetic pesticides cannot. In prior work, we demonstrated that seed treatment with ethanol extract of cell mass of the bacterium *Serratia marcesens* strain N4-5 can control damping-off of cucumber caused by *P. ultimum* as well as the seed treatment pesticide Thiram in certain planting media and soils (Roberts et al. 2014). Preliminary characterization of this natural product from strain N4-5 with thin layer chromatography (TLC) indicated these extracts contained the tripyrrolic red-colored secondary metabolite prodigiosin and the surfactant serrawettin W1 (Roberts et al. 2007). Regions of these TLC plates containing prodigiosin inhibited germination and mycelial growth of *P. ultimum* in vitro. Strain N4-5 also contained *pigC* and *swrW*, genes involved in the biosynthesis of these compounds (Roberts et al. 2007). Here we further characterize ethanol extract of strain N4-5 using liquid chromatography interfaced with a triple quadrupole mass spectrometer (LC/MS–MS) and TLC/MS and demonstrate that purified prodigiosin can control damping-off disease on cucumber caused by *P. ultimum*.

## Materials and methods

### Reagents

All solvents were high performance liquid chromatography (HPLC) grade. Ammonium acetate was LC/MS grade (EMD Millipore, Billerica, MA). HPLC-grade prodigiosin hydrochloride was purchased from Sigma-Aldrich (St. Louis, MO; Cat. No. P0103, CAS No. 82-89-3) to use as a standard for quantification. Water used for analysis was purified by using reverse osmosis and activated carbon. All other reagents used in this study were analytical grade.

### Bacterial and oomycete isolates

Bacterial strains used in this study were maintained at − 80 °C until used. The soil-borne oomycete plant pathogen *P. ultimum* isolate Puzc was maintained on corn meal agar. All bacterial strains and *P. ultimum* isolate Puzc were from the Sustainable Agricultural Systems Laboratory culture collection. *S. marcescens* isolate N4-5 is also accessible at the USDA Agricultural Research Service (ARS) culture collection (nrrl.ncaur.usda.gov) accession B-65519. Unless stated otherwise, kanamycin (Kan), chloramphenicol (Cm), tetracycline (Tc), and ampicillin (Ap) were used at 50 µg/mL, 25 µg/mL, 20 µg/mL, and 50 µg/mL, respectively.

*Serratia marcescens* strain N4-5 was isolated from soil by baiting and identified as *S. marcescens* by fatty acid methyl ester and 16S rRNA DNA sequence analyses (Kobayashi and El-Barrad 1996; Roberts et al. 2007). *S. marcescens* strain Tn246 was constructed by mutagenesis of strain N4-5 with transposon mini-Tn5 Km essentially as described (Roberts et al. 1996). For transposon mutagenesis, overnight shake cultures of *Escherichia coli* S17-1λ *pir* (pUT Km) (DeLorenzo et al. 1990; Herrero et al. 1990) in Luria–Bertani (LB) broth (Miller 1972) plus Ap and Kan, and of strain N4-5 in LB, were washed with sterile distilled water and mated on Nutrient broth agar (NA) plates. The N4-5 derivative strain Tn246, containing a mini-Tn*5* Km insertion, was selected by streaking the mating mixture on NA containing Kan and Tc, followed by verification that the strain was resistant to Kan, Tc, and Cm, and prototrophic on M56 basal salts agar + 0.5% glycerol. Prototrophic strain N4-5 was resistant to Cm and Tc and sensitive to Kan and capable of growth on M56 basal salts agar (Nguyen et al. 1983) + 0.5% glycerol. The auxotrophic strain *E. coli* S17-1 λ *pir* (pUT Km) was resistant to Kan and sensitive to Cm and Tc and not capable of growth on M56 basal salts agar + 0.5% glycerol. Transposon mini-Tn5 Km conferred resistance to Kan in host strains.

A *S. marcescens* N4-5 genome map was used in conjunction with targeted sequencing to detect the location of the mini-Tn5 Km insertion in strain Tn246. Manufacturer (Epicentre Biotechnologies, Madison, WI) supplied primers designed to the 5′ and 3′ flanking regions of mini-Tn5 Km, and oriented to sequence 5′ up-stream and 3′ down-stream, were used (KAN-2 FP-1 Forward Primer 5′-ACCTACAACAAAGCTCTCATCAACC-3′; KAN-2 RP-1 Reverse Primer 5′-GCAATGTAACATCAGAGATTTTGAG-3′). Whole genome sequencing was recently conducted with strain N4-5 (Ferreira et al. 2020) and the genome map can be downloaded from NCBI bioproject PRJNA477367.

### Preparation of ethanol extract from strains N4-5 and Tn246

Cell extracts were prepared from strains N4-5 and Tn246 grown on Peptone Glycerol (PG) agar plates (8 and 80 PG agar plates, respectively) for 3 days at 28 °C. Bacterial cell mass on each PG agar plate was extracted with 10 mL ethanol (Matsuyama et al. 1985), combined and mixed, a 1 mL aliquot of the cell suspension sonicated twice for 30 s each time, and total protein of the cell lysate determined by the method of Bradford (1976). The remaining cell suspension in ethanol was centrifuged at 8000×*g* for 10 min and the supernatant evaporated to dryness with a Rotovap and/or under nitrogen.

### LC/MS–MS characterization of ethanol extract from strains N4-5 and Tn246

Dried ethanol extract, prepared as above, was dissolved in ethanol and further diluted in methanol before analysis. A Waters 2695 LC fitted with an X-bridge C-18 column (150 mm × 2.1 mm i.d., 5 μm) (Waters Corp., Milford, MA) and interfaced with a Micromass Quattro Ultima MS–MS (Waters Corp., Milford, MA) and electrospray ionization (ESI) source was used for analysis. Peak identification was performed in positive full scan mode, scanning from 100 to 1200 *m/z*. Gradient separation was utilized where solvent A was water with 0.1% formic acid and solvent B was acetonitrile with 0.1% formic acid. The solvent gradient program was as follows: initial 90% A:10% B, then linear gradient to 70:30 (A:B) in 10 min; linear gradient to 100% B in 40 min; held at 100% B for 20 min; linear gradient to 90:10 (A:B) in two min; and held for eight min. The flow rate was 0.3 mL/min and typical injections were 10 µL. The column temperature was maintained at 40 °C. Two separate extracts each from strains N4-5 and Tn246 were analyzed, peaks normalized on a total protein basis of the initial cell culture and mean normalized peak quantity with standard deviation determined.

### TLC/MS characterization of prodigiosin and serrawettin W1

Compounds in ethanol extract of strains N4-5 and Tn246, prepared as above, were separated on Silica Gel 60 F254 MS-grade HPTLC plates (EMD Millipore) developed with chloroform: methanol: 5 M ammonium hydroxide (73:23:4) (modified from Matsuyama et al. 1985). Separated compounds on TLC plates were then analyzed by mass spectrometry using an Advion Plate Express plate reader and Advion Expression L compact mass spectrometer (CMS) with an ESI source (Advion, Ithaca, NY). A solvent mixture containing 95% acetonitrile, 5% water, 0.1% acetic acid, and 5 mM ammonium acetate was used to elute the compounds from the TLC plate and carry them to the CMS for ionization. Positive scan mode and a run time of 2 min were used to identify compounds on TLC plates. For detection of surfactant activity, silica gel fines scraped from TLC plates were extracted with sterile distilled water and tested using the drop collapse method (Jain et al. 1991) and rated as in Roberts et al. (2007).

### Prodigiosin purification

Ethanol extract of strain N4-5, prepared as described above, was concentrated to dryness in a Rotovap and dissolved in 3 mL acetone. Prodigiosin was purified from this extract by flash chromatography via elution through a silica (Sigma silica, Cat. No. 236722, 60 Å, 200–245 mesh) prep column with hexane: acetone: ammonium hydroxide (85:15:1; *v:v:v*) as the elution solvent. Orange fractions containing prodigiosin were collected, combined, dried under N_2_, and resuspended in methanol. Purity of the prodigiosin fractions was determined with the LC/MS–MS with an ESI source and X-bridge C-18 reverse phase column (150 mm × 2.1 mm i.d., 5 μm). For this, the diluted aliquots of the prodigiosin fractions were introduced into the C-18 LC column and eluted using gradient separation where solvent A was 0.1% formic acid in water and solvent B was 0.1% formic acid in acetonitrile. The LC operation was the same as described above except the solvent gradient program was terminated after the final gradient reached 100% solvent B when it was brought and held at initial conditions for 8 min. Mass scans of fractions from 100 to 1200 *m/z* were conducted to verify the purity of the prodigiosin preparation (98% pure). Fractions containing only a single peak at 22.6 min, indicative of pure prodigiosin, were combined.

Quantitation of prodigiosin was performed using multiple reaction monitoring (MRM) mode where the collision induced dissociation (CID) transition of parent to daughter ion was 324 *m/z* to 252 *m/z*. This transition was monitored over the time window as prodigiosin was observed to elute under the above conditions, e.g. 22.6 min. The prodigiosin standard was used to create a calibration curve and determine the concentrations of prodigiosin in the ethanol extracts and the purified prodigiosin preparation.

### Control of damping-off of cucumber caused by *P. ultimum*

Experiments to determine suppression of damping-off of cucumber (*Cucumis sativum* cv. Marketmore 76) caused by *P. ultimum* were performed essentially as described (Roberts et al. 1997; 2007). For seed treatment, dried ethanol extract from strain N4-5 was resuspended in 8 mL ethanol, incubated with untreated cucumber seeds for 30 min (8 mL per 6.4 g cucumber seeds), and dried under a laminar flow hood. A sample containing 55.7 mg/mL purified prodigiosin was diluted eightfold with ethanol and used to treat cucumber seeds as above in some experiments. Experiments were also conducted where ethanol extract from N4-5 and Tn246 were normalized on a total protein basis. For this, extract of strain Tn246 was concentrated so that total protein from initial cultures of both strains was similar prior to treating seed. Non-treated cucumber seeds and cucumber seeds incubated in ethanol were used as controls.

To produce inoculum, *P. ultimum* was grown at 25 °C for 3 days, flooded with soil extract (Ayers and Lumsden 1975), and incubated at 25 °C for 7 to 28 days. Sporangia and hyphae of *P. ultimum* from these plates were washed and incorporated into soil-less mix (Pro-Mix PGX, Premier Horticulture, Inc., Quakertown, PA). Soil-less mix, soil-less mix amended with *P. ultimum* or with sterile distilled water, seeds, and soil-less mix amended with *P. ultimum* or sterile distilled water, were added as sequential layers to 6-cm-diameter cups. For each treatment, eight replicate cups were sown with five seeds each and incubated in a growth chamber at 22 °C for 14 days with a 12 h photoperiod. Treatments were arranged in a completely randomized design. Mean plant stand per cup was determined, analysis of variance (ANOVA) carried out, and differences among means estimated using a least-significant difference test protected against Type I experimental error (SAS Institute Inc., Cary, NC). Experiments with purified prodigiosin were performed three times while experiments with strain Tn246 extract were performed four times. Experiments were combined prior to analysis as there was no experiment × treatment interaction with the experiments analyzing purified prodigiosin (*P* = 0.3975) or the experiments comparing extracts from strains N4-5 and Tn246 (*P* = 0.1898).

## Results

### Characterization of N4-5 ethanol extract

**Fig. 1:**
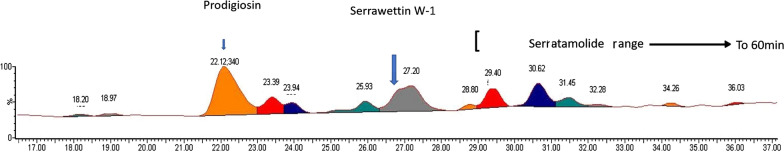
Liquid chromatography interfaced with a triple quadrupole mass spectrometer and electrospray ionization source in full scan positive ion mode analysis of ethanol extract of *Serratia marcescens* isolate N4-5. Ten compounds in addition to prodigiosin and serrawettin W1 were detected by monitoring a mass range from 100-1200 *m/z*. Initially the most abundant chromatographic peaks were identified from this run and then analyzed two more times first to identify their characteristic collision-induced dissociation (CID) fragment ions using a parent > daughter product screening technique and then using selected CID fragment peaks to do a parent > daughter peak quantification method (multiple reaction monitoring) of the extract to measure these areas relative to the areas of prodigiosin for the initially-screened peaks

**Fig. 2:**
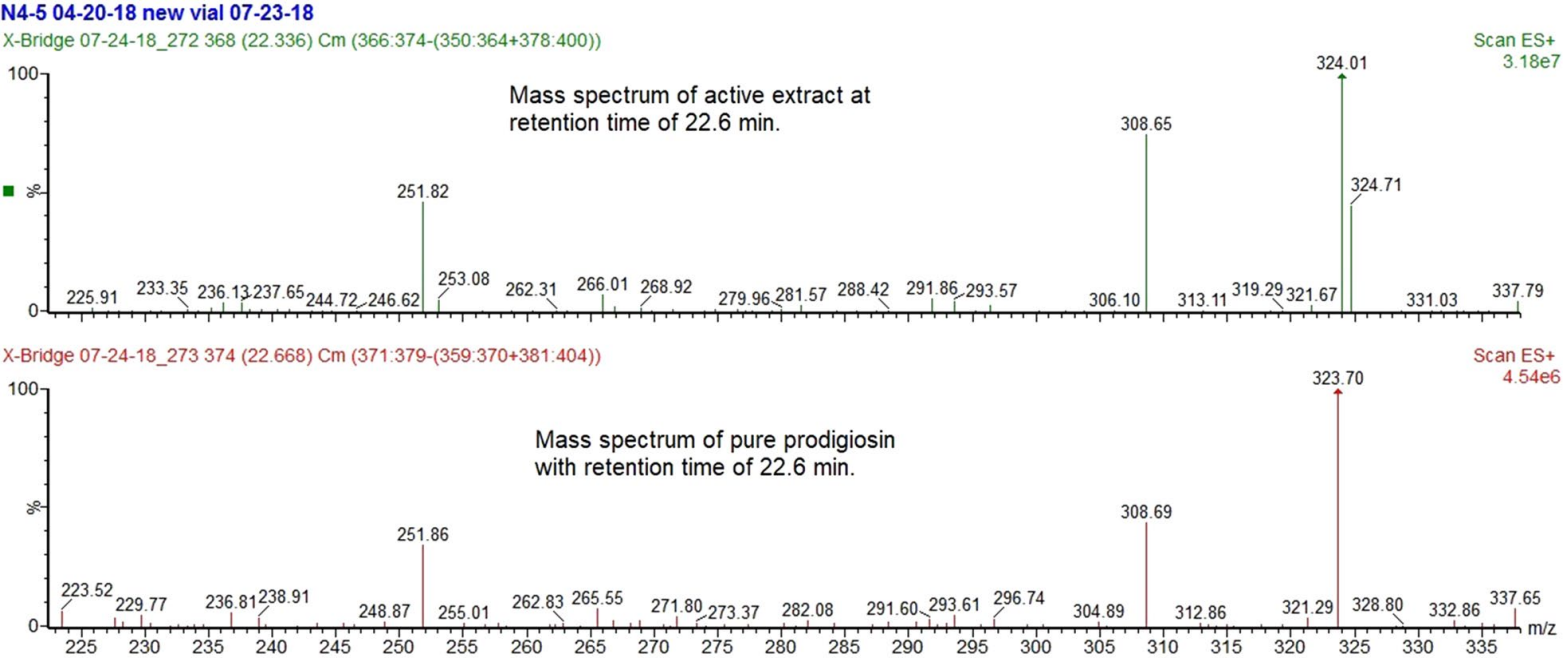
Mass spectra comparison for peaks in two liquid chromatography runs interfaced with a triple quadrupole mass spectrometer and electrospray ionization source in positive ion mode of ethanol extract which eluted at 22.6 min. Top mass spectrometer spectra is for ethanol extract of *Serratia marcescens* N4-5 and lower mass spectrometer spectra is for the same retention time peak for the pure prodigiosin standard

Several compounds were detected in ethanol extract of strain N4-5 (Fig. 1). A large peak was observed that eluted at 22.6 min from the C-18 reverse phase LC column. The major ion in this peak was at 324 *m/z* with a UV absorption maximum of 533 nm, matching characteristics reported for prodigiosin in the literature (Chen 2008; Danyuo et al. 2016). Mass spectrometer fragmentation spectra for this peak also closely matched the positive electrospray mass spectra for the pure prodigiosin standard (Fig. 2). Ethanol extract of strain N4-5 was further characterized regarding prodigiosin with TLC/MS. A fast migrating spot (R_f_ = 0.92) with the characteristic red color of prodigiosin was detected without staining on TLC plates. This spot had a molecular M+H^+^ mass of 324 as determined by mass spectrometry in positive ion mode. The expected molecular mass of prodigoisin was 323.4 (Su et al. 2016).

Several serratamolides, a family of compounds with a similar base structure and surfactant activity (Matsuyama and Nakagawa 1996), were identified in N4-5 ethanol extract (Table 1). The dominant serratamolide was serrawettin W1, which was confirmed by full and selected scan mass spectrometry to match spectral masses published by Thies et al. (2014). These authors also published spectral masses for other serratamolides which were tentatively identified in the extract as M+H^+^ masses of 487, 543 and 571 *m/z*. An additional serratamolide with parent ion M+H^+^ mass of 541 *m/z* was detected which had a spectrum matching a compound reported by Dwivedi et al. (2008). The identities of the serratamolides in the crude extracts were first quality confirmed using a CID product scanning method to verify the CID fragment peaks for the masses listed for these serratomolides in Thies et al. (2014) and Dwivedi et al. (2008). The peak area amounts relative to prodigiosin were determined using the MRM method employing these selected CID parent > daughter fragments and comparing their peak areas relative to MRM peak areas for prodigiosin. Using this method, it was estimated that summing the concentrations of the serratamolids, except serrrawettin W1, represented less than 1% of the prodigiosin detected in N4-5 ethanol extract.

**Table 1:** Compounds identified in ethanol extracts of *Serratia marcescens* strains N4-5 and Tn246

Compound	Mass (*m/z*)	N4-5 Extract	Tn246 Extract
Prodigiosin	324	2,275,312 ± 320,322	1731 ± 1645
Serratamolide	487	8983 ± 1467	552 ± 135
Serratamolide (SW1)	515	653,922 ± 26,249	189,692 ± 16,528
Serratamolide	541	34,499 ± 3051	50,168 ± 9933
Serratamolide	543	66,513 ± 5029	85,575 ± 20,572
Serratamolide	571	3666 ± 579	21,212 ± 3593
Total protein	N/A	1422 ± 325	77 ± 20

Only compounds with a good match to compounds characterized in the literature are listed. Compounds were detected using LC/MS–MS. Values are the mean of two experiments with standard error. Prodigiosin and serratamolide values are area counts per second adjusted for dilution and normalized by protein concentration of cellular lysate [(counts/s)/(µg/mL)]. Total protein concentration (µg/mL) was of cell lysate resulting from sonication of strains N4-5 or Tn246. SW1, serrawettin W1; N/A, not applicable

Surfactant activity fractionated into three zones when N4-5 ethanol extract was analyzed with TLC. TLC fines extracted with sterile distilled water from a slow migrating region (R_f_ = 0.17) of the TLC plates had weak surfactant activity while TLC fines from two fast-migrating zones (R_f_ = 0.71–0.79; R_f_ = 0.87) had strong surfactant activity; with the strongest surfactant activity being detected in the zone R_f_ = 0.71–0.79. No mass was detected by mass spectrometry in positive or negative ion mode in the slow migrating zone (R_f_ = 0.17) with weak surfactant activity. A compound with M+H^+^ mass 537.4 was detected in the zone with the greatest surfactant activity (R_f_ = 0.71–0.79) in positive ion mode. This compound had the expected mass of serrawettin W1 (MW = 514.6) complexed with sodium (MW = 22.9). A compound with molecular M+H^+^ mass 569.4 was detected in positive ion mode in the other fast migrating zone with surfactant activity (R_f_ = 0.87).

Additional compounds with M+H^+^ masses of 488, 536, 684, 834, 906, and 908 *m/z* were detected inconsistently in LC/MS–MS runs with ethanol extract of strain N4-5. The compounds with M+H^+^ masses of 684 and 536 *m/z* had CID fragmentation spectra similar with compounds detected in *Serratia* sp. in prior studies (Dwivedi et al. 2008; Thies et al. 2014).

### Characterization of strain Tn246 and Tn246 ethanol extract

**Fig. 3:**
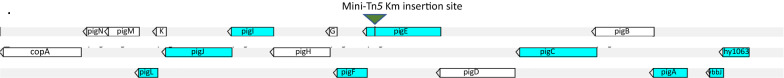
Transposon insertion site within *pig* cluster of *Serratia marcescens* strain Tn246. The *pig* genes are responsible for biosynthesis of prodigiosin. Mini-Tn*5* km, transposon mini-Tn*5* km

DNA sequence analysis of the genome of strain Tn246 identified a single mini-Tn5 Km insertion within *pigE* (Fig. 3). *pigE* encodes an 853 amino acid protein with 100% identity to the *Serratia* multispecies aminotransferase class III-fold pyridoxal phosphate-dependent enzyme, which has been shown to be involved in the biosynthesis of prodigiosin (Su et al. 2016). As expected with this mutation, ethanol extract of strain Tn246 had no MS peak at the 22.6 min retention time indicative of prodigiosin and minimal UV absorbance at 533 nm. Trace amounts of prodigiosin are indicated in Table 1. However, these trace amounts were 1300-fold lower than in N4-5 extract and possibly due to the large correction factor employed to normalize the strain N4-5 and Tn246 data on a total protein basis.

Ethanol extract from strain Tn246 contained all serratamolides detected in ethanol extract from strain N4-5. Serrawettin W1 (M+H^+^ mass 515.3) was present but at levels 3.4-fold lower than in N4-5 extract after normalization of data on a total protein basis. Serratamolides with M+H^+^ masses of 487, 541, 543, and 571 were detected at levels 16-fold lower, 1.5-fold higher, 1.3-fold higher, and 5.8-fold higher than in N4-5 extract. Surfactant activity was detected in ethanol extract of strain Tn246 in TLC fines from the region of the TLC plate containing serrawettin W1 (R_f_ = 0.71–0.79). MS analysis of this region of the plate detected a compound with M+H^+^ mass of 537.4 in positive ion mode. As with ethanol extract from strain N4-5 this compound had the expected mass of serrawettin W1 complexed with sodium. Surfactant activity was not detected with TLC fines from other regions of the TLC plates. As with N4-5 ethanol extract, compounds with M+H^+^ masses of 488, 536, 684, 834, 906, and 908 *m/z* were detected inconsistently in LC/MS–MS runs with the Tn246 ethanol extract.

### Role of prodigiosin in control of *P. ultimum* damping-off of cucumber

Prodigiosin, purified from ethanol extract of strain N4-5, provided control of damping-off of cucumber (Table 2). Plant stand was significantly greater than the non-treated and ethanol-only controls with the treatment containing cucumber seeds treated with an eightfold dilution of a 55.7 mg/mL concentration of the purified prodigiosin preparation (approximately 35.6 µg/seed) when 30 sporangia/cm^3^ soil-less mix inoculum was applied. There was no disease control with this treatment when 50 sporangia/cm^3^ soil-less mix inoculum was applied. The positive control, ethanol extract of strain N4-5, provided control at both the 30 and 50 sporangia/cm^3^ inoculum levels. It should be noted that the concentration of prodigiosin in the N4-5 extract treatment used in these experiments was not determined.

**Table 2:** Suppression of damping-off of cucumber caused by *Pythium ultimum* in soil-less mix with prodigiosin purified from ethanol extract of *Serratia marcescens* N4-5

Treatment^a^	Mean plant stand per pot at different *P. ultimum* infestation levels^b^			
	0	10	30	50
Non-treated seed	4.75 A	4.00 A → D	2.17 E	1.46 EF
Ethanol-only	4.67 AB	3.33 CD	0.79 F	1.13 EF
N4-5 ethanol extract	4.42 AB	4.21 ABC	3.42 CD	3.96 A → D
Purified prodigiosin	4.67 AB	3.71 BCD	3.21 CD	1.58 EF

^a^See “Materials and methods” for a description of treatments. Non-treated, no treatment was applied; ethanol-only, seeds were treated with ethanol only; N4-5 ethanol extract, seeds were treated with ethanol extract of strain N4-5; purified prodigiosin, seeds were treated with purified prodigiosin in ethanol

^b^Values were the mean of three experiments (*n* = 3) expressed as mean plant stand per pot. Results were combined prior to analysis as there was no significant experiment × treatment effect (*P* = 0.3975). Treatments in all experiments contained eight replicate pots, each containing five seeds. Numbers followed by the same letter were not significantly different (*P* ≤ 0.05) as determined by a protected least significant difference test. Least significant difference was 0.97. *P. ultimum* was added at 0, 10, 30, and 50 sporangia/cm^3^ soil-less mix inoculum

In a second set of experiments, ethanol extract of strain Tn246, which was shown to be devoid of prodigiosin, did not control damping-off of cucumber (Table 3). Seed treatment with ethanol extract of strain Tn246 was similar with the non-treated and ethanol-only controls at all levels of inoculum of the pathogen. In contrast, seed treatment with the N4-5 extract diluted 1/3 with ethanol provided disease control at the highest level of inoculum. Plant stand associated with this treatment was significantly greater than that associated with the Tn246 extract at this highest level of inoculum. The 1/3 dilution of the N4-5 ethanol extract had similar total protein in the originating cell mass to the ethanol extract from strain Tn246. Seed treated with a more dilute preparation (¼ dilution) of N4-5 ethanol extract also provided disease control resulting in a plant stand significantly greater than the non-treated and ethanol-only controls at the 30 sporangia/cm^3^ and 50 sporangia/cm^3^ inoculum levels of the pathogen. Plant stand associated with this treatment was significantly greater than that with the Tn246 treatment at both these levels of inoculum. Seed treatment with the ethanol-only control resulted in a plant stand similar with that of the non-treated control at all levels of pathogen inoculum.

**Table 3:** Suppression of damping-off of cucumber caused by *Pythium ultimum* in soil-less mix with ethanol extract from *Serratia marcescens* strains N4-5 and Tn246 normalized on a total protein basis

Treatment^a^	Mean plant stand per pot at different *P. ultimum* infestation levels^b^			
	0	10	30	50
Non-treated seed	4.84 AB	3.78 DEF	2.16 KLM	1.50 MN
Ethanol-only	4.94 A	3.63 EFG	2.56 I → L	1.13 N
1X N4-5 ethanol extract	4.59 A → D	4.25 A → E	3.50 E → H	3.81 C → F
1/3X N4-5 ethanol extract	4.88 AB	4.03 B → E	2.66 H → K	3.13 F→J
1/4X N4-5 ethanol extract	4.88 AB	4.66 A → D	3.44 E → I	2.88 G → K
Tn246 ethanol extract	4.68 ABC	3.41 E → I	2.44 JKL	1.75 LMN

^a^See “Materials and methods” for a description of treatments. Non-treated seed; no treatment was applied. Ethanol-only, seeds were treated with ethanol. N4-5 ethanol extract, seeds were treated with ethanol extract of strain N4-5. Tn246 ethanol extract, seeds were treated with ethanol extract of strain Tn246

^b^Values were the mean of four experiments (*n* = 4) expressed as mean plant stand per pot. Results were combined prior to analysis as there was no significant experiment × treatment effect (*P* = 0.1898). Treatments in all  experiments contained eight replicate pots, each containing five seeds. Numbers followed by the same letter were not significantly different (*P* ≤ 0.05) as determined by a protected least significant difference test. Least significant difference was 0.68. *P. ultimum* was added at 0, 10, 30, and 50 sporangia/cm^3^ soil-less mix inoculum

## Discussion

The tripyrrolic compound prodigiosin was confirmed to be present in ethanol extract of *S. marcescens* N4-5 in experiments reported here. A spot with the characteristic red coloration, R_f_, and mass of prodigiosin was detected by TLC/MS in these ethanol extracts. In separate LC/MS–MS experiments with this extract a compound with the expected mass and fragmentation pattern for prodigiosin was detected. We also demonstrate here that this compound prodigiosin plays a role in control of damping-off of cucumber caused by the oomycete plant pathogen *P. ultimum.* Cucumber seed treated with purified prodigiosin resulted in a plant stand that was significantly greater than nontreated cucumber seed in the presence of some levels of inoculum of *P. ultimum*. The purified prodigiosin preparation had only the single peak eluting at 22.6 min, characteristic of prodigiosin, when analyzed by LC/MS–MS indicating that no other compounds were in the purified preparation. Consistent with this, cucumber seed treated with the ethanol extract of mutant strain Tn246, which was devoid of prodigiosin, did not control disease caused by this pathogen. All other compounds detected in ethanol extract of strain N4-5 were detected in the ethanol extract of Tn246 at relatively similar levels.

Prodigiosin has been reported to be a bioactive compound with anti-oomycetal, anti-fungal, anti-bacterial, anti-protozoal, and anti-insectal properties as well as having immunosuppressive and anti-tumor activities (Danevčič et al. 2016; Demain 1995; Domröse et al. 2015; Lapenda et al. 2015; Parani and Saha 2008; Roberts et al. 2007; Someya et al. 2001; Suryawanshi et al. 2017; Tsuji et al. 1990; 1992; Williams and Quadri 1980). These anti-microbial and other activities have been attributed to membrane potential alteration and damage, phototoxicity, and formation of reactive oxygen species (Busschaert and Gale 2013; Darshan and Manonmani 2016; Suryawanshi et al. 2017; Wang et al. 2013). However, this is the first report demonstrating that prodigiosin can control a plant disease.

The compound serrawettin W1 was also confirmed to be present in ethanol extract of *S. marcescens* N4-5 in experiments reported here. A spot with the characteristic R_f_ and mass of serrawettin W1 was detected by TLC/MS and the expected mass and fragmentation pattern detected with LC/MS–MS. Serrawettin W1 has been reported to have several bioactivities including antimicrobial activity against oomycetes and bacteria, and surfactant and wetting activity (Kadouri and Shanks 2013; Matsuyama et al. 1985; Shemyakin et al. 1965; Strobel et al. 2005). At least one additional surfactant has been detected in ethanol extract from this strain, as well as numerous serratamolides, in experiments reported here and elsewhere (Roberts et al. 2007). Compounds detected inconsistently in ethanol extract of strain N4-5 with M+H^+^ masses of 834, 906, and 908 *m/z* were similar in structure to serrawettin W2. The serrawettin W2 group of compounds are composed of a fatty acid connected cyclically with five amino acids and have surfactant activity (Su et al. 2016). None of the compounds in ethanol extract of strain N4-5, however, exactly matched compounds discussed by Su et al. (2016) but each had similar ESI positive mass spectrometer fragments suggesting a cyclic compound composed of a fatty acid and two or more amino acid residues.

The roles of serrawettin W1 and the serratamolides/surfactants in controlling damping-off of cucumber are not known. However, synergistic activities of surfactants with diverse antibiotics have been reported (Ben Kheder et al. 2017; D’aes et al. 2010; Perneel et al. 2008; Rossi et al. 2016; Sotirova et al. 2012; Williamson et al. 2008; Yin 2014). Previous studies have suggested that prodigiosin produced by another strain of *Serratia* had anti-biotic activity only when in combination with a surfactant produced by this strain (Williamson et al. 2008). Additionally, combinatorial anti-biotic effects have been reported with prodigiosin and serrawettin W1 as well as other surfactants (Hage-Hülsmann et al. 2018). These surfactants may also help prodigiosin adhere to the seed (Roberts et al. unpublished). Future experiments will be directed at the role of these compounds when applied as seed treatments in plant disease control.
